# Variation in Seed Metabolites between Two Indica Rice Accessions Differing in Seed Longevity

**DOI:** 10.3390/plants9091237

**Published:** 2020-09-19

**Authors:** Jae-Sung Lee, Fiona R. Hay

**Affiliations:** 1 T.T. Chang Genetic Resources Center, Strategic Innovation Platform, International Rice Research Institute, Los Baños, College, Laguna 4031, Philippines; JS.Lee@irri.org; 2 Department of Agroecology, Aarhus University, Forsøgsvej 1, 4200 Slagelse, Denmark

**Keywords:** QTOF mass spectrometry, rice, seed metabolites, seed longevity

## Abstract

For a better understanding of germination after seed storage, metabolite profiling was conducted using hybrid triple quadrupole time-of-flight (QTOF) mass spectrometry. After moisture content (MC) equilibration, seeds of “WAS170” (short-lived) and “IR65483” (long-lived) were stored at 10.9% MC and 45 °C. Samples for metabolite analysis were taken after 0 and 20 days of storage. Among 288 metabolites, two flavonoids (kaempferide and quercetin-3-arabinoside), one amino acid (S-sulfocysteine) and one sugar (D-glucose) increased in “IR65483” seeds after storage but were not detected in “WAS170” seeds. Based on the genome sequence database, we identified clear allelic differences with non-synonymous mutations on the six flavonol synthase genes regulating the accumulation of kaempferol- and quercetin-metabolites. On the other hand, two metabolites (thiamine monophosphate and harmaline) increased in short-lived seeds after storage; these metabolites could be potential biochemical indicators of seed deterioration.

## 1. Introduction

To support the efficient conservation of rice genetic resources, in previous research we have sought to better understand the variation in seed longevity within a diverse panel of Indica rice varieties [1]. We were able to identify varieties that produce seeds that are relatively short-lived and varieties that produce seeds that are relatively long-lived, and identify genes that may play a role in determining relative seed longevity. Consistent with other studies, one longevity mechanism may be related to the ability to repair macromolecules in the early stages of germination [2,3]. Genes related to root development, sugar metabolism and scavenging of reactive oxygen species were also significantly correlated with at least one longevity parameter. In relation to this, we [4,5] and others [6,7] have also suggested a role for vitamin E, an important anti-oxidant, in the seed longevity response. In the current study, we took a metabolomics approach to understand the underlying physiological differences in seeds that are short- or long-lived in experimental storage, if they are removed from experimental storage after the same period.

Metabolomics is a key approach to understand any biological mechanism of interest. Compared with conventional analytic methods such as high-performance liquid chromatography (HPLC), rapid separation liquid chromatography (RSLC) or gas chromatography-mass spectrometry (GC-MS), quadrupole time-of-flight (QTOF) mass spectrometry provides higher resolving power to separate a large number of individual metabolites [8]. Here, we used a hybrid triple QTOF system to profile and compare seed metabolites of two Indica rice accessions differing in seed longevity in order to gain a better understanding of ageing tolerance from a physiological perspective.

## 2. Results and Discussion

Based on data presented in Lee et al. [1], seeds of “WAS170” [9] are relatively short-lived in storage, with a *p*_50_ (time taken for viability to fall to 50%) of 19.37 days when stored at 10.9% MC and 45 °C; seeds of “IR65483” [10] are relatively long-lived with a *p*_50_ under the same storage conditions of 45.43 days (seed viability after 20 days of storage was 97%). A total of 288 metabolites comprising 42 amino acids, two ammonium compounds, four aromatic compounds, one capsaicinoid, one fatty acid, 21 flavonoids, one heterocyclic compound, five hormones, four lipids, 29 nucleobases, two organic acids, 41 organic compounds, one phosphoric acid, one polyol compound, 12 sugars, two sugar alcohols, one theophylline, 15 vitamins and 103 unknown peaks were detected in the two accessions. Metabolites varied between accessions and treatments (control and stored (10.9% MC and 45 °C) seeds) (Table 1; Supplementary Table S1).

After 20 days of storage followed by 24 h imbition in water, the number of metabolites increased and reduced was 145 and 132, respectively, in seeds of “WAS170” and 174 and 108, respectively, in seeds of “IR65483”. Four metabolites, kaempferide, quercetin-3-arabinoside, S-sulfocysteine and D-glucose, increased in “IR65483” seeds (with greater longevity) after 20-days storage but were not detected in “WAS170” seeds (Table 1). Kaempferol- and quercetin-metabolites are flavonols which act as antioxidants, scavenging reactive oxygen species induced by biotic or abiotic stresses such as pathogens, UV radiation and heat [11]. Flavonols are known to increase in mature seeds of Arabidopsis but reduce in some other species, e.g., buckwheat [12,13]. Physiological roles of kaempferide and quercetin-3-arabinoside in seed germination after storage need to be explored further. Based on the genome sequence database (Rice Genome Annotation Project) [14], we identified 23 flavonol synthase genes regulating the accumulation of kaempferol- and quercetin-metabolites [15].

To assess allelic differences of the two accessions in the region of those genes, we used the Rice SNP-Seek Database which provides information on the 18 million single nucleotide polymorphisms (SNP) base set for 3000 diverse rice accessions including “WAS170” and “IR65483” [16,17]. SNP markers in the region of 23 flavonol synthase genes were extracted and clear allelic differences between the two accessions were observed for the six flavonol synthase genes (Table 2). Based on premature stop codons located at the SNP positions listed in Table 2 [16], we speculate that non-synonymous mutations on LOC_Os04g57160 in “IR65483” might positively regulate the conversion of dihydrokaempferol or dihydroquercetin into kaempferol or quercetin, whereas mutations on LOC_Os01g25010 and LOC_Os11g25060 in “WAS170” negatively regulate the conversion process. Further studies are needed to determine variation in flavonol content due to functional mutations, in particular, SNP markers. These markers were not significantly associated with seed longevity traits in our previous genome-wide association (GWA) analysis using a large indica rice panel. This does not mean that the results are contradictory. Rather, the results obtained from the different approaches (metabolite analysis and GWA) should be considered complementary [18]. For example, S-sulfocysteine plays an important role in chloroplast functions such as the maintenance of chlorophyll content and photosynthetic activities [19]. L-cysteine, derived from S-sulfocysteine through the sulphate pathway, is one of the main ways in which glutathione is synthesized. Glutathione is a key antioxidant that prevents oxidized intracellular conditions in seeds subjected to storage conditions [20]. Although the role of sugar in seed longevity has not been clearly defined [21], some studies suggest that sugars may enhance seed longevity via specific physico-chemical mechanisms such as limiting molecular mobility and transforming the cytoplasm into a glassy state [22].

Two metabolites, thiamine monophosphate and harmaline, increased in “WAS170” (short-lived) seeds after storage (Table 1). They could be seed ageing-metabolites and potential biochemical indicators of seed deterioration. Gołda et al. [23] reported that thiamine-binding activity and total thiamine content in seeds rapidly reduced during germination in maize, oat, faba bean and garden pea. Therefore, accumulation of thiamine monophosphate after seed storage may reflect imbalanced germination processes in short-lived seeds. Three unknown metabolites highly increased after storage in both accessions (Table 1). Isonicotinic acid is an isomer of niacin, a water-soluble vitamin B similar to thiamine. Prodanov et al. [24] reported that niacin content significantly increased during the germination of legumes seeds. However, a physiological function of this metabolite with respect to seed longevity has not yet been studied.

We would like to recommend that metabolite analysis using advanced QTOF mass spectrometry may provide enhanced metabolic information as compared with single mass spectrometry and hence could speed up the discovery of physiological mechanisms for traits such as seed longevity. The identified SNP markers regulating flavonols and consequent seed germination after storage can promote both seed longevity research and routine operations in genebanks [1,25,26]. Based on the presence or absence of particular markers associated with seed longevity traits, genebank managers can adjust the interval for seed viability monitoring and regeneration. Those markers could also be used in breeding programs to improve the seed longevity and seed-lot vigour of rice varieties widely grown in hot and humid regions, while the metabolites themselves have potential application as biomarkers to follow the seed ageing process during storage [27].

## 3. Materials and Methods

Seed lots and longevity data used in this study are as described in Lee et al. [1]. Briefly, the seeds of two *Oryza sativa* Indica-group accessions used in this metabolite study, “IR65483” [10] and “WAS170” [9], were produced on the Zeigler Experiment Station of the International Rice Research Institute in the 2015 dry-season. Both accessions flowered at 130 days after sowing (DAS) and seeds were harvested at 45 days after flowering (DAF). A portion of the seeds was used to determine their longevity parameters [1], the remaining seeds were kept in aluminium foil bags at −20 °C (for approximately one month) and used for metabolite analysis. The bags were removed from storage and allowed to equilibrate to room temperature before opening. Samples of the seeds of the two accessions were equilibrated at 60% RH, 25 °C for one week and then transferred to laminated aluminium foil bags that were immediately heat-sealed and placed at 45 °C. After 0 and 20 days storage, respectively, the palea and lemma were removed and the caryopses (it was not possible to use only embryonic tissue due to the small size of the embryo in rice seeds) placed as three replicates of 50 seeds on two layers of Whatman No. 1 filter paper in 90 mm-diameter Petri dishes with 7 mL distilled water. Based on RNA expression analysis for “WAS170” and “IR65483” (Lee et al., unpublished data), most of the genes involving seed metabolism were not active after the standard post-harvest drying procedure. After 24 h imbibition to stimulate metabolism in seed tissues, samples were finely ground using liquid nitrogen and then freeze-dried and stored at −20 °C. Samples were sent by airmail in an ice pack to the National Instrumentation Center for Environmental Management (NICEM), Seoul National University, Korea for metabolite analysis. An amount of 200 mg of sample was incubated in 20 mL 70% methanol at 25 °C for 2 h. After centrifugation, the supernatant sample was filtered through a 0.2-μm membrane filter and then diluted (1/100) by 70% methanol. Three internal replicates per sample were used to increase the accuracy in the analysis. Metabolite identification was conducted by TripleTOF 5600 (AB SCIEX, Concord, ON, CA) connected to Ultimate 3000 RSLC HPLC system (Thermo Fisher Scientific Inc., MA, USA) following Yong et al. [28]. A reversed-phase column (Kinetex 1.7 um C18, 100A, 100 × 2.1 mm, Phenomenex) was used for the LC separation. The data were analyzed by Analyst TF v1.7, PeakView v2.2, MarkerView v1.2.1.1 and Elements v1.3.1. Metabolites were then classified into different chemical categories. The mean and standard error of the peak intensities of individual metabolites were calculated. To compare the peak intensities between the two accessions at the same storage period (i.e., 0 or 20 days) or between storage periods within each accession, t-tests were carried out using Genstat Release 20.1 (VSN International Ltd., Hemel Hempstead, UK).

## Figures and Tables

**Table 1:** Metabolites that showed the greatest variation between treatments during germination of “WAS170” (short-lived) and “IR65483” (long-lived) rice seeds after 0 (“control”) and 20 days storage (“stored”) at 10.9% moisture content (MC) and 45 °C. Metabolites were determined by quadrupole time-of-flight (QTOF) mass spectrometry. The values (mean ± s.e. peak intensities for three ground samples of 50 seeds for each accession × storage treatment) are peak intensities from an extracted ion chromatogram.

No	Metabolite	Class	“WAS170” (Control)	“IR65483” (Control)	“WAS170” (Stored)	“IR65483” (Stored)†
**Increased accumulation during germination in long-lived seeds**						
1	Kaempferide	Flavonoid	0.00 ± 0.00	0.00 ± 0.00	0.00 ± 0.00	188.20 ± 43.56
2	Quercetin-3-Arabinoside	Flavonoid	0.00 ± 0.00	0.00 ± 0.00	0.00 ± 0.00	144.09 ± 13.16
3	S-Sulfocysteine	Amino acid	0.00 ± 0.00	28.72 ± 4.92 a	0.00 ± 0.00	59.58 ± 1.97 b
4	D-Glucose	Sugar	0.00 ± 0.00	0.00 ± 0.00	0.00 ± 0.00	45.84 ± 17.16
**Increased accumulation during germination in short-lived seeds**						
5	Thiamine monophosphate	Vitamin	0.00 ± 0.00	0.00 ± 0.00	91.87 ± 10.97	0.00 ± 0.00
6	Harmaline	Organic compound	0.00 ± 0.00	0.00 ± 0.00	67.08 ± 10.46	0.00 ± 0.00
**Increased accumulation during germination in seeds of both varieties**						
7	442.2834/7.79	Unknown	0.00 ± 0.00	0.00 ± 0.00	23321.3 ± 4132.7 A	20705.0 ± 2378.5 A
8	442.7854/7.77	Unknown	0.00 ± 0.00	0.00 ± 0.00	12357.9 ± 2221.6 A	12046.5 ± 1129.5 A
9	474.2805/7.54	Unknown	0.00 ± 0.00	0.00 ± 0.00	3206.9 ± 609.1 A	3509.7 ± 581.4 A
10	Isonicotinic acid	Organic compound	406.25 ± 74.70 b A	234.07 ± 16.24 b A	2487.1 ± 129.0 a A	1748.0 ± 134.8 a B
11	Erythrose 4-phosphate	Phosphoric acid	0.00 ± 0.00	0.00 ± 0.00	131.36 ± 3.59 B	166.61 ± 4.50 A
12	Kaempferol-3-Glucuronide	Flavonoid	0.00 ± 0.00	0.00 ± 0.00	29.19 ± 1.40 B	66.75 ± 1.57 A
13	Poncirin	Flavonoid	0.00 ± 0.00	0.00 ± 0.00	24.57 ± 12.70 A	14.79 ± 3.81 A
14	Folic acid	Vitamin	0.00 ± 0.00	0.00 ± 0.00	17.62 ± 7.38 A	7.03 ± 3.98 A

Comparisons of peak intensities between the two accessions after the same seed storage period or between the two storage periods for the same accession were made when the intensities were > 0 for both samples. Values with different lower- or upper-case letters are significantly different. The lower-case letters show the results of comparisons within an accession for different storage periods; upper-case letters show the results of comparisons between accessions for the same storage period (*P* < 0.05).

**Table 2:** Allelic differences between rice accessions “WAS170” (short-lived) and “IR65483” (long-lived) on six flavonol synthase genes regulating flavonols accumulation. Red font indicates non-synonymous mutations.

Accession	Locus ID	LOC_Os01g25010			LOC_Os04g57160	LOC_Os06g06720	LOC_Os08g37456	LOC_Os09g18390	LOC_Os11g25060			
	Position	14102569	14102621	14102690	34062284	3145594	23725210	11290674	14286630	14286868	14290631	14290791
“WAS170”		**C**	**A**	**G**	A	T	T	**C**	**A**	**G**	**T**	**G**
“IR65483”		A	C	T	**G**	**C**	**A**	G	C	T	A	C

## Supplementary Materials

The following are available online at https://www.mdpi.com/2223-7747/9/9/1237/s1, Table S1: Seed metabolite profiling using two indica varieties, ‘WAS170’ and ‘IR65483’ with low and high seed longevity, respectively. Values highlighted in green indicate more than 10-fold changes for the comparisons indicated.
