# Supplementary material for: Variation in Seed Metabolites between Two Indica Rice Accessions Differing in Seed Longevity
Source: Plants (Basel). 2020 Sep 19;9(9):1237. doi: 10.3390/plants9091237 (PMC7569829; doi:10.3390/plants9091237)
Supplement: Supplementary file 1 [file plants-09-01237-s001.pdf]

Supplementary Table S1. Seed metabolite profiling using two indica varieties, 'WAS170' and 'IR65483' with low and high seed longevity, respectively.

No	Peak Name	Class	m/z	Ret. Time	'WAS170' (control)	s.e.	'IR65483' (control)	s.e.	'WAS170' (stored)	s.e.	'IR65483' (stored)	s.e.	Difference after storage ('WAS170')	Difference after storage ('IR65483')
1	2-Aminobutyric acid	Amino acid	104.07	0.99	1225.90	129.52	2399.47	213.26	2775.47	425.64	4762.61	282.73	1549.57	2363.14
2	Agmatine	Amino acid	131.13	0.92	37.16	3.53	26.20	3.77	31.90	2.94	38.94	2.05	-5.26	12.74
3	Ala	Amino acid	161.09	11.82	223.74	45.36	198.45	10.88	198.18	22.62	167.93	14.52	-25.56	-30.52
4	Aminocyclopropane-1-carboxylic acid	Amino acid	102.06	1.00	106.00	9.28	200.84	13.46	79.88	13.34	90.50	18.12	-26.12	-110.34
5	Aminovaleric acid	Amino acid	118.09	1.06	12615.94	427.03	15010.81	1398.29	8913.71	585.73	9530.74	730.74	-3702.23	-5480.07
6	Arginine	Amino acid	175.12	0.96	3936.10	756.88	7572.47	1251.25	3579.66	316.78	5008.70	352.62	-356.44	-2563.77
7	Beta-Alanine	Amino acid	90.06	0.99	114.78	31.32	150.27	16.76	269.27	42.38	372.57	28.80	154.49	222.30
8	Beta-homoisoleucine	Amino acid	146.12	1.04	64.12	7.75	77.26	14.09	30.13	11.33	19.05	19.05	-33.98	-58.21
9	Beta-homolysine	Amino acid	161.13	13.12	172.71	11.55	223.26	4.98	235.61	14.31	277.05	6.92	62.90	53.79
10	Beta-homothreonine	Amino acid	134.08	1.00	86.06	9.15	101.35	8.05	52.19	10.92	75.84	6.16	-33.87	-25.52
11	Beta-homotryptophan	Amino acid	219.11	3.21	0.00	0.00	98.48	17.03	0.00	0.00	0.00	0.00	0.00	-98.48
12	Carnosine	Amino acid	227.11	13.10	518.06	110.31	680.82	44.98	500.68	71.76	484.81	23.71	-17.39	-196.01
13	Cystathionine	Amino acid	223.07	8.14	72.55	31.87	40.38	10.50	108.87	41.27	50.55	13.52	36.32	10.17
14	Cysteinylglycine	Amino acid	179.05	1.05	164.09	32.01	387.47	22.15	196.67	13.66	213.99	9.42	32.57	-173.48
15	Diaminopimelic acid	Amino acid	191.10	15.31	634.26	51.61	523.51	18.53	779.93	33.10	713.70	18.02	145.67	190.19
16	Ethionine	Amino acid	164.07	0.99	75.57	3.70	306.87	38.30	0.00	0.00	31.85	4.00	-75.57	-275.02
17	Glutamine	Amino acid	147.08	1.00	962.68	36.03	1802.67	161.15	1138.21	49.05	1095.22	33.39	175.53	-707.45
18	Gly-Gly	Amino acid	133.06	0.99	1399.97	8.70	1594.40	174.97	1380.52	62.41	1345.34	13.40	-19.45	-249.07
19	Histidine	Amino acid	156.08	0.95	1531.71	110.27	1963.85	80.33	776.90	78.59	901.48	94.98	-754.82	-1062.37
			170.09	1.00	100.12	25.97	59.36	10.98	156.15	11.92	96.28	19.91	56.03	36.92
			sum		1631.83		2023.21		933.05		997.75		-698.79	-1025.46
20	Isoleucine	Amino acid	132.10	1.30	17620.83	1885.81	43781.91	5075.83	16004.83	2430.30	20623.74	1850.76	-1615.99	-23158.16
21	Kynurenine	Amino acid	225.09	4.37	131.55	24.81	89.76	31.68	0.00	0.00	0.00	0.00	-131.55	-89.76
			225.09	5.56	0.00	0.00	0.00	0.00	66.22	8.78	72.30	4.61	66.22	72.30
			sum		131.55		89.76		66.22		72.30		-65.33	-17.47
22	Leucylleucyltyrosine	Amino acid	408.25	10.15	64.95	0.39	142.01	8.39	67.11	3.27	153.41	4.84	2.16	11.39
23	Lysine	Amino acid	147.11	0.92	280.24	141.34	646.25	69.59	597.80	26.39	648.63	5.37	317.56	2.38
24	Methionine	Amino acid	150.06	1.07	428.82	41.07	983.84	56.18	555.66	156.41	608.19	62.44	126.84	-375.65
25	Methoxy-3-carbaldehyde	Amino acid	176.07	5.79	597.53	28.92	563.94	5.15	918.58	14.26	858.76	41.22	321.05	294.82
26	N-Acetyl-DL-aspartic acid	Amino acid	176.06	5.79	25.93	12.09	49.04	0.84	61.58	1.69	67.71	1.51	35.66	18.67
27	N-alpha-Acetyl-L-ornithine	Amino acid	175.11	0.96	1150.73	210.14	2365.29	393.99	1056.77	72.08	1534.40	101.80	-93.96	-830.89
28	O-Acetyl-L-homoserine	Amino acid	162.08	1.04	292.02	11.85	409.13	23.07	334.37	14.86	362.92	27.56	42.34	-46.21
29	O-acetyl-L-serine	Amino acid	148.06	1.01	5805.07	440.90	9507.08	215.05	3753.95	413.29	4919.14	322.05	-2051.12	-4587.94
30	Phenylalanine	Amino acid	198.08	1.06	163.19	2.94	227.91	10.05	131.10	2.82	139.23	6.14	-32.09	-88.67
			166.09	1.60	10384.49	1155.52	29418.46	4414.34	8150.56	970.48	11225.11	480.07	-2233.92	-18193.35
			sum		10547.68		29646.37		8281.67		11364.34		-2266.01	-18282.02
31	Proline	Amino acid	116.07	1.05	531.13	34.55	1194.62	110.43	1166.99	132.89	1746.62	23.88	635.86	552.00
32	Pyroglutamic acid	Amino acid	130.05	1.00	1096.14	37.95	2039.72	124.02	1092.07	29.59	1181.80	20.00	-4.07	-857.92
33	Quinic acid	Amino acid	193.07	4.99	126.96	10.21	349.25	26.99	0.00	0.00	0.00	0.00	-126.96	-349.25
34	S-(5'-Adenosyl)-L-methionine	Amino acid	399.14	0.99	17.60	2.81	29.56	1.19	17.60	3.02	11.29	0.86	0.00	-18.27
35	Saccharopine	Amino acid	277.14	0.99	71.80	3.61	90.28	8.32	0.00	0.00	11.43	11.43	-71.80	-78.85
			277.14	5.35	0.00	0.00	0.00	0.00	103.94	5.16	79.56	9.15	103.94	79.56
			sum		71.80		90.28		103.94		90.99		32.14	0.71
36	S-Adenosyl-L-homocysteine	Amino acid	385.13	1.05	83.60	1.77	89.04	7.67	22.96	0.98	38.42	1.57	-60.64	-50.62
37	S-Sulfocysteine	Amino acid	201.98	0.90	0.00	0.00	28.72	4.92	0.00	0.00	59.58	1.97	0.00	30.86
38	Threonine	Amino acid	120.07	1.00	652.66	53.64	1144.75	163.65	900.64	100.02	964.57	37.02	247.98	-180.18
39	trans-4-Hydroxy-L-proline	Amino acid	132.07	1.01	148.99	17.31	172.00	29.19	135.94	17.39	219.56	16.91	-13.05	47.56
40	Tryptophane	Amino acid	205.10	2.80	6482.73	424.53	4849.55	300.07	8737.18	1143.84	2024.09	103.80	2254.45	-2825.47
41	Tyrosine	Amino acid	182.08	1.29	4138.83	599.86	7212.82	802.33	4008.52	502.56	5512.51	312.50	-130.30	-1700.30
42	Glucosamine	Amino acid/sugar	180.09	1.11	442.58	8.40	674.27	42.31	401.78	31.79	377.25	4.31	-40.80	-297.02

43	Carnitine	Ammonium compound	162.11	1.02	619.77	23.23	649.90	28.64	305.14	2.46	365.75	15.72	-314.63	-284.15
44	O-Acetyl-L-carnitine	Ammonium compound	205.13	15.31	141.09	25.83	104.79	16.45	189.33	3.87	171.24	6.22	48.23	66.45
45	Anthranilic acid	Aromatic compound	138.06	1.06	2557.51	278.39	2143.03	103.38	3502.26	429.95	2409.31	57.77	944.75	266.28
46	Methyl Dihydrojasmonate	Aromatic compound	227.16	9.33	60.26	9.60	68.88	4.12	0.00	0.00	0.00	0.00	-60.26	-68.88
47	Oxypurinol	Aromatic compound	153.04	0.90	82.05	2.71	165.03	4.38	140.96	10.03	251.55	13.12	58.91	86.52
48	Sinapic acid	Aromatic compound	225.08	4.37	295.18	44.91	282.79	36.08	0.00	0.00	0.00	0.00	-295.18	-282.79
			225.08	5.54	0.00	0.00	0.00	0.00	147.25	14.82	185.49	9.34	147.25	185.49
			sum		295.18		282.79		147.25		185.49		-147.93	-97.31
49	Capsaicin	Capsaicinoids	306.21	7.28	19.75	19.75	24.07	19.30	0.00	0.00	0.00	0.00	-19.75	-24.07
50	Gamma-linolenic acid	Fatty acid	279.23	10.92	0.00	0.00	0.00	0.00	14228.20	2050.34	0.00	0.00	14228.20	0.00
			279.23	12.62	6690.91	155.51	10275.79	252.19	0.00	0.00	14218.69	287.72	-6690.91	3942.90
			sum		6690.91		10275.79		14228.20		14218.69		7537.29	3942.90
51	Apigenin-7-O-neohesperidoside	Flavonoid	579.17	5.93	0.00	0.00	208.57	77.02	0.00	0.00	85.98	85.98	0.00	-122.59
52	Baicalin	Flavonoid-like	447.09	14.27	13.22	13.22	0.00	0.00	93.31	83.12	0.00	0.00	80.09	0.00
53	Eriodictyol-7-O-glucoside	Flavonoid	451.12	1.05	297.98	2.63	205.57	42.47	303.14	18.82	356.44	36.36	5.16	150.87
54	Hydroflavone	Flavonoid	225.09	9.95	11.99	0.77	42.93	12.41	0.00	0.00	33.34	7.67	-11.99	-9.59
55	isorhamnetin-3-O-rutinoside	Flavonoid	625.18	5.48	93.34	14.76	149.58	46.78	0.00	0.00	191.64	25.13	-93.34	42.06
56	Kaempferide	Flavonoid	301.07	8.20	0.00	0.00	0.00	0.00	0.00	0.00	188.20	43.56	0.00	188.20
57	Kaempferol-3-Glucoside-2''-Rhamnoside-7-R	Flavonoid	741.22	15.66	504.06	101.82	535.08	44.33	345.09	96.12	300.00	30.21	-158.96	-235.09
58	Kaempferol-3-Glucuronide	Flavonoid	463.09	0.99	0.00	0.00	0.00	0.00	29.19	1.40	66.75	1.57	29.19	66.75
59	kaempferol-3-O-rutinoside	Flavonoid	595.17	5.10	227.25	227.25	1493.00	70.41	79.66	79.66	1010.23	195.90	-147.59	-482.77
60	Kaempferol-3-Rhamnoside-4''-Rhamnoside-7	Flavonoid	725.23	3.99	0.00	0.00	0.00	0.00	84.49	15.47	31.19	21.35	84.49	31.19
			725.23	6.19	43.45	10.22	23.79	12.24	0.00	0.00	0.00	0.00	-43.45	-23.79
			sum		43.45		23.79		84.49		31.19		41.04	7.39
61	Neodiosmin	Flavonoid	609.18	6.06	58.86	4.10	150.07	30.63	15.45	15.45	90.60	14.06	-43.41	-59.48
62	Oenin	Flavonoid	494.14	6.35	30.03	30.03	114.05	33.49	0.00	0.00	0.00	0.00	-30.03	-114.05
63	Poncirin	Flavonoid	595.20	1.01	0.00	0.00	0.00	0.00	24.57	12.70	14.79	3.81	24.57	14.79
64	Quercetin-3-Arabinoside	Flavonoid	435.09	4.44	0.00	0.00	0.00	0.00	0.00	0.00	144.09	13.16	0.00	144.09
65	Quercetin-3-O-beta-glucopyranosyl-7-O-alph	Flavonoid	611.16	18.21	964.38	278.65	806.83	36.98	1017.87	259.52	614.79	37.13	53.49	-192.03
66	Trihydroxyflavanone	Flavonoid	273.08	0.97	61.38	10.20	163.56	12.73	64.37	7.92	147.27	10.73	2.99	-16.29
67	Naringenin-7-O-glucoside	Flavonoid	435.13	0.97	137.48	14.03	132.37	7.41	186.63	15.01	158.77	13.29	49.14	26.40
68	Catechin	Flavonoid-phenolic	291.09	1.00	192.30	29.49	735.49	50.11	103.51	22.45	245.07	46.06	-88.79	-490.42
69	Glucopyranosyl sinapate	Flavonoid-phenolic	387.13	4.57	300.09	48.95	265.77	37.43	169.48	43.72	132.03	26.90	-130.61	-133.74
70	Procyanidin B1	Flavonoid-polyphenol	579.15	15.78	194.21	29.94	209.35	7.01	149.15	36.47	172.94	24.43	-45.07	-36.41
71	Procyanidin C1	Flavonoid-polyphenol	867.21	0.99	14.23	1.11	67.85	4.09	3.66	3.66	7.75	4.61	-10.57	-60.10
			867.21	3.84	0.00	0.00	0.00	0.00	28.09	8.62	6.85	3.42	28.09	6.85
			sum		14.23		67.85		31.74		14.60		17.51	-53.25
72	Thiazoleethanol	Heterocyclic compounds	144.05	0.94	135.39	7.26	88.15	4.73	136.43	3.90	82.10	5.32	1.04	-6.05
73	3-Indolylacetonitrile	Hormone	157.08	0.99	114.48	8.11	144.06	8.52	24.80	11.20	45.38	11.92	-89.68	-98.68
74	Gibberellin	Hormone	333.17	9.25	78.78	10.06	88.56	3.02	96.12	2.59	94.75	1.09	17.34	6.18
75	Indole-3-acetaldehyde	Hormone	160.08	1.72	816.75	38.67	1408.32	82.69	567.84	130.34	770.59	102.49	-248.91	-637.73
76	Indole-3-carboxyaldehyde	Hormone	146.06	2.80	629.50	89.41	527.72	29.54	689.58	121.75	160.33	9.66	60.08	-367.40
77	Indole-3-carboxylic acid	Hormone	162.06	1.34	103.80	12.50	8.75	8.75	383.31	53.40	433.28	55.75	279.51	424.53
			162.06	4.98	0.00	0.00	102.04	21.80	0.00	0.00	0.00	0.00	0.00	-102.04
			sum		103.80		110.79		383.31		433.28		279.51	322.49
78	Glycero-3-Phosphocholine	Lipid	413.25	15.17	2020.24	189.78	2306.45	61.99	2266.82	128.80	2041.24	59.50	246.58	-265.22
			441.29	15.88	1693.25	213.56	1499.47	195.65	1832.96	142.01	1880.70	138.20	139.71	381.23
			sum		3713.49		3805.92		4099.78		3921.93		386.29	116.02
79	O-Phosphocholine	Lipid	185.08	1.05	195.88	5.52	166.21	7.07	222.84	17.81	222.00	19.63	26.97	55.80
80	Prostaglandin E1	Lipid	355.25	12.44	39.43	7.58	82.52	6.71	19.03	7.84	65.76	12.56	-20.40	-16.76
81	sn-Glycero-3-phosphocholine	Lipid	259.12	1.04	2978.77	189.22	2762.87	120.01	1698.19	39.34	1309.02	53.49	-1280.58	-1453.85
82	3-Methyladenine	Nucleobase	150.08	1.00	34.77	7.66	48.72	6.28	76.20	5.70	92.35	8.03	41.44	43.64
83	Adenine	Nucleobase	136.06	1.29	216.64	23.52	302.67	8.87	242.05	5.44	351.45	27.95	25.41	48.78
84	Adenosine	Nucleobase	268.10	1.31	1218.68	84.60	1701.44	77.29	1390.17	47.72	2205.53	173.41	171.49	504.09

85	Adenosine 5'-diphosphate	Nucleobase	428.04	1.18	0.00	0.00	142.23	54.86	0.00	0.00	0.00	0.00	0.00	-142.23
86	Adenosine 5'-diphospho-glucose	Nucleobase	590.09	14.01	105.20	28.67	95.56	7.54	56.65	29.08	54.57	24.97	-48.55	-40.99
87	Aminoimidazole-4-carboxamide-1-beta-D-rib	Nucleobase	339.07	10.30	0.00	0.00	0.00	0.00	0.00	0.00	0.00	0.00	0.00	0.00
88	Beta-Nicotinamide adenine dinucleotide	Nucleobase	664.12	1.05	223.86	18.23	237.63	11.23	114.75	17.69	107.96	27.16	-109.11	-129.67
89	Beta-Nicotinamide mononucleotide	Nucleobase	335.06	1.02	139.38	3.77	116.39	4.62	113.57	5.46	98.48	3.70	-25.80	-17.90
90	Cytidine	Nucleobase	244.09	0.99	84.62	2.56	79.92	3.63	65.03	7.10	74.22	9.43	-19.59	-5.70
91	Deoxyadenosine	Nucleobase	252.11	1.02	70.89	10.34	87.81	8.94	154.19	8.93	146.46	15.50	83.29	58.65
92	Deoxyadenosine-5'-monophosphate	Nucleobase	332.08	1.04	44.91	0.89	46.08	8.46	47.54	5.00	15.45	8.17	2.63	-30.62
			332.08	4.44	0.00	0.00	0.00	0.00	0.00	0.00	59.72	30.71	0.00	59.72
			sum		44.91		46.08		47.54		75.17		2.63	29.10
93	Deoxyguanosine 5'-monophosphate	Nucleobase	348.07	1.05	225.99	12.41	239.06	28.12	393.84	21.78	63.40	26.20	167.85	-175.66
94	Deoxyuridine-5'-monophosphate	Nucleobase	309.05	0.97	75.50	5.59	70.33	0.56	61.46	5.61	87.71	6.84	-14.04	17.38
95	Guanine	Nucleobase	152.06	1.35	290.62	24.34	441.03	31.33	372.37	30.37	457.91	44.95	81.75	16.87
96	Guanosine	Nucleobase	284.10	1.35	252.59	5.54	400.69	24.26	322.74	13.65	453.53	44.63	70.15	52.84
97	Guanosine 5'-diphosphoglucose	Nucleobase	606.08	14.74	148.08	39.18	266.40	20.89	219.52	45.44	220.65	5.25	71.44	-45.75
98	Guanosine-3',5'-cyclic monophosphate	Nucleobase	346.05	1.01	12.60	1.41	48.98	11.16	4.73	2.46	13.18	6.67	-7.87	-35.80
99	Methylthioadenosine	Nucleobase	298.10	2.65	801.56	102.88	1110.77	49.64	403.59	18.43	749.88	19.75	-397.97	-360.89
100	N-6-(delta-2-Isopentenyl)adenosine	Nucleobase	336.17	13.13	142.17	5.64	161.10	5.73	174.18	15.17	199.21	8.06	32.01	38.11
101	NADH	Nucleobase	666.13	15.16	1414.70	233.95	1654.36	69.52	1453.67	236.65	1420.09	124.98	38.96	-234.27
102	Thymidine-5'-monophosphate	Nucleobase	323.06	0.97	112.25	8.53	86.52	4.73	69.65	0.66	60.58	3.95	-42.60	-25.94
103	Thymine	Nucleobase	127.05	1.03	159.51	6.39	199.37	3.51	223.56	8.52	169.54	9.89	64.05	-29.82
104	trans-Zeatin	Nucleobase	220.12	1.69	6638.92	162.01	4454.07	232.31	7238.06	393.27	6078.89	269.44	599.14	1624.82
105	Uridine	Nucleobase	245.08	9.10	61.58	11.78	65.72	2.83	94.43	2.90	110.74	6.38	32.84	45.01
106	Uridine 5'-diphosphate	Nucleobase	405.01	1.11	127.48	8.77	225.66	26.70	7.07	3.66	6.56	6.56	-120.41	-219.10
107	Uridine 5'-diphospho-D-glucose	Nucleobase	567.06	14.04	459.91	68.27	487.62	12.35	407.29	60.84	392.86	37.08	-52.62	-94.77
108	Uridine 5'-diphosphoglucuronic acid	Nucleobase	581.04	14.03	657.99	78.57	843.50	21.93	726.76	93.89	786.09	37.88	68.77	-57.41
109	Uridine 5'-diphospho-N-acetylgalactosamine	Nucleobase	608.09	14.74	1291.84	237.35	1536.54	41.77	1424.88	220.91	1401.52	128.60	133.04	-135.02
110	Urocanic acid	Nucleobase	139.05	1.04	90.06	11.20	81.39	16.90	255.76	15.96	216.79	14.55	165.70	135.41
111	Methylsuccinic acid	Organic acid	133.05	0.99	937.09	7.02	1081.27	120.58	944.62	36.91	923.76	19.81	7.53	-157.51
112	Tartaric acid	Organic acid	151.02	0.89	310.55	13.18	808.56	19.39	571.20	57.18	1133.82	11.85	260.65	325.26
113	Acetylcholine	Organic compound	147.13	13.12	71.80	14.92	99.99	6.56	114.26	8.17	119.76	3.80	42.46	19.78
114	Allantoin	Organic compound	159.05	5.50	297.55	57.96	399.03	55.46	228.38	34.97	253.66	17.39	-69.17	-145.37
115	Benzamidine	Organic compound	121.08	1.64	61.44	33.20	258.73	33.34	63.50	31.99	91.74	9.32	2.06	-166.99
116	Cholate	Organic compound	409.29	15.30	185.43	22.60	193.22	2.08	204.13	15.58	141.01	30.40	18.70	-52.22
117	cis-Aconitic acid	Organic compound	175.02	1.27	410.97	49.01	934.03	38.45	293.00	2.02	706.81	74.14	-117.97	-227.22
118	Citric acid	Organic compound	193.03	1.27	191.03	7.79	381.84	24.19	136.73	7.13	342.93	18.49	-54.29	-38.91
119	Citrulline	Organic compound	176.10	1.01	92.81	3.55	100.09	5.22	120.89	10.92	109.87	6.20	28.08	9.78
120	Cyanidin-3,5-di-O-glucoside	Organic compound	612.17	18.22	6071.76	2274.41	3658.59	167.41	5135.89	1521.37	2578.22	156.49	-935.87	-1080.37
121	Cyanidin-3-O-(2''-O-beta-xylopyranosyl-beta-	Organic compound	582.16	1.01	0.00	0.00	11.94	2.30	5.47	2.74	5.14	2.79	5.47	-6.80
122	Cyanidin-3-O-(2''-O-beta-xylopyranosyl-beta-	Organic compound	744.21	15.66	177.49	33.32	164.24	9.10	132.70	17.59	121.65	15.70	-44.79	-42.59
123	Deoxycholate	Organic compound	393.30	9.79	341.79	108.52	488.35	141.07	273.51	5.32	232.72	17.08	-68.28	-255.63
124	Dimethoxycinnamic acid	Organic compound	209.08	4.99	79.20	28.83	199.76	14.50	191.12	6.69	261.60	1.36	111.92	61.84
125	Farnesol	Organic compound	223.21	13.69	63.43	4.86	45.75	14.68	155.35	7.20	132.38	11.95	91.92	86.63
126	Gluconasturtiin	Organic compound	424.07	1.00	27.45	4.31	9.91	1.85	30.39	6.80	26.32	7.68	2.94	16.41
127	Glutarate	Organic compound	163.06	1.04	1379.02	69.55	1838.97	121.85	1646.33	25.70	1495.31	98.42	267.31	-343.67
128	Harmaline	Organic compound	215.12	7.53	0.00	0.00	0.00	0.00	67.08	10.46	0.00	0.00	67.08	0.00
129	Hinokitiol	Organic compound	165.09	13.09	156.38	78.24	0.00	0.00	138.72	69.47	18.46	18.46	-17.66	18.46
130	Hydroxy benzoic acid	Organic compound	155.03	10.79	83.46	30.79	125.09	7.49	58.27	29.20	87.92	2.15	-25.18	-37.17
131	Hydroxybenzoate	Organic compound	139.04	1.04	23.61	12.84	50.05	24.18	228.74	17.21	228.17	26.03	205.13	178.12
132	Hypotaurine	Organic compound	110.03	0.90	0.00	0.00	42.64	1.79	29.47	8.85	69.36	3.92	29.47	26.72
133	Isonicotinic acid	Organic compound	124.04	1.28	406.25	74.70	234.07	16.24	2487.12	129.04	1748.03	134.82	2080.88	1513.96
134	Leupeptin	Organic compound	427.30	11.65	45.83	20.05	145.62	12.98	205.65	10.91	188.63	18.78	159.82	43.02
135	Lipoic acid	Organic compound	207.05	4.52	108.15	26.54	145.73	16.67	0.00	0.00	0.00	0.00	-108.15	-145.73
			207.05	5.54	0.00	0.00	0.00	0.00	91.32	7.63	135.36	3.77	91.32	135.36

			sum		108.15		145.73		91.32		135.36		-16.83	-10.37
136	Mailc acid	Organic compound	149.04	4.70	430.21	45.24	396.44	17.54	503.53	116.86	346.17	22.05	73.32	-50.26
137	Mandelic acid	Organic compound	153.05	4.71	286.42	34.99	389.85	21.91	315.13	19.18	378.98	12.93	28.71	-10.87
138	Methoxycinnamic acid	Organic compound	179.07	4.93	4571.72	482.61	840.87	130.31	356.64	22.99	461.18	18.58	-4215.08	-379.69
			195.07	5.38	330.06	19.95	0.00	0.00	485.69	59.01	475.04	19.42	155.63	475.04
			sum		4901.78		840.87		842.33		936.22		-4059.45	95.35
139	Methylglutaric acid	Organic compound	147.07	1.00	642.99	21.63	1153.86	103.08	692.61	40.65	668.01	36.79	49.62	-485.85
140	m-Hydroxycinnamic acid	Organic compound	165.05	1.30	2215.41	241.65	3712.96	437.60	2231.77	279.92	2912.67	144.43	16.36	-800.29
141	N1-Acetylspermine	Organic compound	245.23	13.12	2372.92	166.64	2764.71	66.38	3245.25	154.12	3821.36	70.12	872.33	1056.65
142	Nicotine	Organic compound	163.12	4.90	86.48	18.50	118.35	7.94	71.61	5.71	79.80	2.26	-14.87	-38.55
143	Nitrilotriethanol	Organic compound	150.11	0.98	324.02	25.16	202.29	10.45	246.91	6.92	178.19	19.10	-77.11	-24.10
144	Pipecolinic acid	Organic compound	130.09	0.94	583.07	69.14	920.19	39.58	740.73	19.28	1001.24	51.64	157.66	81.05
145	Purine	Organic compound	204.12	1.05	205.10	25.82	143.74	20.21	0.00	0.00	6.12	6.12	-205.10	-137.62
146	Pyrrolidine	Organic compound	177.14	10.77	117.84	3.48	116.56	6.04	0.00	0.00	0.00	0.00	-117.84	-116.56
147	Rosmarinic acid	Organic compound	361.09	6.57	62.33	3.01	78.20	5.10	0.00	0.00	65.33	9.64	-62.33	-12.87
148	Safranine	Organic compound	316.17	10.62	44.68	6.42	49.58	10.17	0.00	0.00	0.00	0.00	-44.68	-49.58
149	Solasodine	Organic compound	414.34	11.90	94.34	21.36	42.02	21.01	69.93	43.60	78.91	15.10	-24.41	36.90
			414.34	13.11	66.91	6.25	157.89	36.10	95.16	49.23	53.84	30.85	28.25	-104.06
			sum		161.25		199.91		165.09		132.75		3.84	-67.16
150	Syringaldehyde	Organic compound	183.07	7.22	108.26	8.08	111.35	3.01	248.44	25.43	205.14	22.24	140.18	93.79
151	trans-Cinnamic acid	Organic compound	149.06	1.01	106.79	26.13	79.96	40.04	55.40	6.28	57.12	21.72	-51.38	-22.84
			149.06	1.61	10.63	10.63	184.22	105.17	0.00	0.00	40.00	23.11	-10.63	-144.22
			sum		117.42		264.18		55.40		97.12		-62.01	-167.06
152	Umbelliferone	Organic compound	177.05	4.14	0.00	0.00	1473.03	757.26	0.00	0.00	0.00	0.00	0.00	-1473.03
			177.05	5.38	1988.33	113.28	1182.61	431.67	2627.34	370.29	2602.32	181.15	639.01	1419.72
			sum		1988.33		2655.64		2627.34		2602.32		639.01	-53.32
153	Glutathione	Organic compound	613.16	18.21	185.16	62.01	139.14	6.48	182.23	47.31	102.72	2.11	-2.93	-36.43
154	Erythrose 4-phosphate	Phosphoric acid	201.02	2.54	0.00	0.00	0.00	0.00	131.36	3.59	166.61	4.50	131.36	166.61
155	Glycero-3-Phosphate	Polyol compound	537.36	14.07	1077.10	94.74	1292.35	29.97	1148.08	123.85	1234.26	60.68	70.99	-58.09
156	Cellotriose	Sugar	505.18	1.02	1606.75	47.09	836.05	55.72	1230.36	25.98	663.29	11.20	-376.39	-172.77
157	D-Glucose	Sugar	341.00	1.05	0.00	0.00	0.00	0.00	0.00	0.00	45.84	17.16	0.00	45.84
158	Glucose-6-phosphate	Sugar	261.04	1.03	2506.07	236.34	2365.00	55.17	6362.51	151.59	8442.54	317.79	3856.43	6077.54
159	Hesperidin	Sugar	611.20	18.22	1518.60	758.58	522.10	13.20	879.19	316.96	363.74	49.37	-639.41	-158.36
160	N-acetylneuraminic acid	Sugar	310.11	1.01	19.58	5.41	36.51	9.34	26.82	6.43	30.25	10.45	7.24	-6.26
161	peonidin-3-o-beta-d-glucopyranoside	Sugar	464.13	12.98	0.00	0.00	0.00	0.00	39.23	39.23	0.00	0.00	39.23	0.00
162	Peonidin-3-O-beta-galactopyranoside	Sugar	464.13	1.03	12.58	12.58	97.15	6.02	49.72	27.18	119.36	12.36	37.14	22.21
163	Rhamnose	Sugar	165.08	1.02	274.90	60.48	45.00	23.75	149.15	8.94	161.53	27.16	-125.75	116.53
164	Sissotrin	Sugar	447.13	5.56	27.08	9.77	78.01	12.27	9.70	9.70	87.94	9.26	-17.38	9.93
			447.13	16.71	0.00	0.00	0.00	0.00	0.00	0.00	0.00	0.00	0.00	0.00
			sum		27.08		78.01		9.70		87.94		-17.38	9.93
165	Sucrose	Sugar	343.12	1.07	4148.66	68.87	3895.29	173.79	4589.63	114.94	3126.23	70.35	440.98	-769.06
166	UDP-beta-L-rhamnose	Sugar	551.07	14.06	73.40	8.79	66.83	3.44	55.60	4.13	53.32	5.82	-17.79	-13.51
167	UDP-xylose	Sugar	537.05	14.07	343.09	56.48	351.51	13.59	278.61	36.44	257.30	25.12	-64.49	-94.21
168	Iditol	Sugar alcohol	183.09	1.30	1069.87	174.65	1383.20	224.73	872.52	202.40	1021.40	48.67	-197.35	-361.80
169	Maltitol	Sugar alcohol	345.14	1.02	0.00	0.00	0.00	0.00	14.28	10.76	0.00	0.00	14.28	0.00
			345.14	6.58	79.06	4.39	88.21	3.49	0.00	0.00	15.55	8.21	-79.06	-72.66
			sum		79.06		88.21		14.28		15.55		-64.78	-72.66
170	Methylurate	Theophylline	197.07	4.94	186.02	15.18	0.00	0.00	0.00	0.00	0.00	0.00	-186.02	0.00
171	4-Pyridoxate	Vitamin	184.06	1.05	1421.52	57.51	1330.28	25.02	1473.65	64.90	1633.02	159.55	52.12	302.75
172	Alpha-tocotrienol	Vitamin	425.34	14.80	1411.73	243.90	2002.10	91.49	1735.19	290.75	1807.60	49.69	323.46	-194.49
173	Calciferol	Vitamin	397.35	13.92	222.42	81.72	230.39	5.94	202.84	103.60	137.64	54.35	-19.57	-92.74
			397.35	16.50	0.00	0.00	0.00	0.00	0.00	0.00	118.79	60.42	0.00	118.79
			sum		222.42		230.39		202.84		256.44		-19.57	26.05
174	Choline	Vitamin-like	105.11	1.00	302.29	4.07	290.93	10.37	589.09	50.75	621.54	32.61	286.80	330.62

175	Delta-Tocotrienol	Vitamin	397.31	13.92	261.00	130.50	167.52	7.39	297.02	148.84	0.00	0.00	36.03	-167.52
			397.31	14.82	149.93	99.94	423.55	69.30	129.84	129.84	635.35	30.74	-20.09	211.80
			sum		410.93		591.06		426.86		635.35		15.94	44.29
176	Folic acid	Vitamin	442.15	7.83	0.00	0.00	0.00	0.00	17.62	7.38	7.03	3.98	17.62	7.03
177	Gamma-tocotrienol	Vitamin	411.33	15.31	3478.20	357.88	2698.53	52.32	4538.83	74.29	3945.16	145.78	1060.63	1246.63
178	Niacinamide	Vitamin	123.06	1.28	147.56	8.33	226.61	78.18	116.32	21.27	120.65	10.73	-31.24	-105.96
179	Nicotinic acid mono nucleotide	Vitamin	337.06	10.31	0.00	0.00	0.00	0.00	14.52	14.52	0.00	0.00	14.52	0.00
180	Pyridoxal	Vitamin	168.07	1.02	51.53	4.97	58.28	2.87	80.92	7.99	72.09	1.50	29.39	13.81
181	Pyridoxine	Vitamin	170.08	1.03	54.53	4.09	40.96	3.82	174.30	26.41	138.26	4.12	119.76	97.31
182	Retinoic acid	Vitamin	301.22	9.83	13775.27	1236.29	21224.33	1413.97	1265.19	204.82	242.54	57.38	-12510.08	-20981.79
183	Riboflavin-5'-monophosphate	Vitamin	457.11	0.98	80.40	6.37	49.54	2.50	68.16	3.04	71.58	4.32	-12.24	22.04
184	Thiamine	Vitamin	266.12	0.94	212.95	4.68	135.25	18.51	301.27	11.22	146.81	14.54	88.32	11.56
185	Thiamine monophosphate	Vitamin	346.09	4.21	0.00	0.00	0.00	0.00	91.87	10.97	0.00	0.00	91.87	0.00
186	110.0085 / 0.89	Unknown	110.01	0.89	1486.74	61.70	3817.24	58.17	2855.09	215.78	5414.56	107.97	1368.35	1597.32
187	188.0700 / 2.84	Unknown	188.07	2.79	12411.79	1018.03	7120.58	828.83	14867.24	2377.44	2686.93	401.15	2455.45	-4433.64
188	201.1842 / 14.09	Unknown	201.18	14.10	1871.95	666.66	2124.32	1480.58	851.01	523.65	1501.21	568.74	-1020.95	-623.11
189	213.1450 / 6.83	Unknown	213.15	6.83	6296.94	922.35	7487.71	266.19	6147.54	730.48	7000.56	98.24	-149.39	-487.16
190	218.2103 / 7.33	Unknown	218.21	7.33	3239.65	247.31	5727.06	633.81	491.38	119.38	560.65	108.82	-2748.28	-5166.40
191	235.1433 / 3.22	Unknown	235.14	3.20	8320.78	805.35	21345.79	4120.43	844.74	172.38	2772.80	492.07	-7476.04	-18572.99
192	246.2417 / 8.71	Unknown	246.24	8.71	9251.90	402.56	13394.58	1456.86	1465.05	94.36	2057.96	348.49	-7786.85	-11336.62
193	258.2776 / 10.36	Unknown	258.28	10.36	18746.48	1240.09	22508.27	2369.82	1129.18	418.41	1264.25	164.05	-17617.30	-21244.02
194	262.2368 / 7.98	Unknown	262.24	7.98	16413.34	648.16	25700.05	3281.39	562.58	124.82	695.01	177.02	-15850.76	-25005.04
195	263.2354 / 13.11	Unknown	263.24	13.12	17658.77	1242.05	22322.17	487.66	25282.03	1162.83	30929.49	571.25	7623.26	8607.31
196	265.2513 / 13.66	Unknown	265.25	13.69	7112.03	49.52	5812.80	156.24	16808.38	1210.53	15277.25	354.90	9696.35	9464.46
197	271.2732 / 8.91	Unknown	271.27	8.91	3841.25	183.11	6066.60	334.48	100.31	16.12	262.65	15.01	-3740.94	-5803.94
198	274.2732 / 9.84	Unknown	274.27	9.85	5479.44	744.62	7406.98	783.16	1177.48	115.19	1803.75	418.74	-4301.97	-5603.24
199	281.2459 / 13.11	Unknown	281.25	13.11	24255.89	1591.23	30773.49	795.11	35110.43	1714.51	43507.21	838.75	10854.53	12733.72
200	283.2619 / 13.70	Unknown	283.26	13.69	13716.33	67.07	11302.22	204.20	32252.40	2441.83	29173.49	481.26	18536.08	17871.27
201	286.3095 / 11.41	Unknown	286.31	11.42	15270.84	1854.17	16361.61	821.04	1065.44	62.88	1033.60	202.72	-14205.40	-15328.01
202	290.2672 / 9.18	Unknown	290.27	9.18	26273.91	3494.47	38514.29	4032.17	1959.08	778.03	2369.99	619.67	-24314.83	-36144.30
203	295.2623 / 14.70	Unknown	295.26	14.70	12134.82	655.74	10272.97	657.22	5122.88	497.45	5793.81	715.39	-7011.94	-4479.16
204	297.2779 / 15.24	Unknown	297.28	15.26	9254.64	525.10	6660.84	381.36	4231.85	455.94	5462.52	628.38	-5022.80	-1198.32
205	298.2730 / 10.11	Unknown	298.27	10.12	1895.60	75.58	4436.38	274.60	2901.96	45.08	6026.65	70.11	1006.36	1590.27
206	299.3045 / 10.02	Unknown	299.30	10.03	24219.01	1048.10	17671.90	929.57	1346.19	153.63	2341.38	219.57	-22872.81	-15330.52
207	303.1949 / 8.60	Unknown	303.19	8.61	5169.84	276.91	3482.57	416.99	116.86	9.84	49.87	2.87	-5052.98	-3432.70
208	303.2306 / 9.04	Unknown	303.23	9.04	5628.37	432.61	6419.05	246.65	147.85	16.29	58.49	5.93	-5480.52	-6360.56
209	304.2841 / 9.38	Unknown	304.28	9.38	3267.69	1114.62	2691.11	766.56	132.92	46.18	12.69	6.44	-3134.77	-2678.42
210	313.2726 / 12.79	Unknown	313.27	12.79	8024.32	380.30	12515.34	224.22	8137.04	521.25	15630.40	231.78	112.71	3115.06
211	315.1941 / 10.64	Unknown	315.19	10.63	24127.36	1594.29	25431.93	2261.90	733.44	126.63	404.32	37.19	-23393.92	-25027.61
212	316.2836 / 9.72	Unknown	316.28	9.73	1946.25	149.91	4104.69	320.80	3017.16	60.73	6642.14	210.13	1070.90	2537.46
213	317.2098 / 9.91	Unknown	317.21	9.91	8766.27	447.19	11507.93	569.04	326.33	140.80	111.80	23.83	-8439.94	-11396.14
214	318.2988 / 10.22	Unknown	318.30	10.22	23651.82	2534.49	33777.95	2410.89	13407.96	220.42	24779.50	891.90	-10243.86	-8998.45
215	319.2251 / 9.07	Unknown	319.23	9.07	16349.42	1098.09	24004.54	951.80	255.72	118.60	109.70	9.02	-16093.70	-23894.84
216	323.1380 / 6.58	Unknown	323.14	6.57	6321.76	324.93	9233.21	148.17	378.82	59.59	1650.72	219.11	-5942.94	-7582.48
217	331.1887 / 9.70	Unknown	331.19	9.71	13592.04	890.46	18764.23	667.39	900.60	109.03	679.28	47.78	-12691.44	-18084.96
218	331.2830 / 12.79	Unknown	331.28	12.79	6483.89	225.10	9945.26	154.56	6533.55	476.89	12063.59	263.92	49.66	2118.33
219	337.1765 / 10.60	Unknown	337.18	10.63	14790.34	582.68	14681.52	1005.12	1299.08	218.10	781.92	77.58	-13491.26	-13899.59
220	337.2369 / 7.08	Unknown	337.24	7.08	3985.63	124.16	872.69	145.97	831.29	70.47	139.40	8.23	-3154.34	-733.30
221	338.3409 / 14.77	Unknown	338.34	14.77	10842.99	5949.78	12023.18	2737.21	15618.07	3786.44	13504.57	3569.39	4775.07	1481.40
222	346.2117 / 5.20	Unknown	346.21	5.20	5416.16	242.55	6615.50	132.39	5011.19	598.58	5229.95	644.76	-404.97	-1385.54
223	353.1707 / 9.68	Unknown	353.17	9.70	10919.72	447.43	13263.60	221.90	1702.97	144.59	1365.19	80.62	-9216.74	-11898.42
224	353.3513 / 11.37	Unknown	353.35	11.37	6348.31	237.50	3337.84	147.34	848.62	153.15	535.02	109.83	-5499.69	-2802.81
225	360.2267 / 5.65	Unknown	360.23	5.66	7297.33	553.93	10026.73	448.61	6879.38	1053.98	7932.62	891.85	-417.95	-2094.10
226	381.0792 / 0.98	Unknown	381.08	0.98	24493.48	77.47	47337.89	1492.08	41579.87	1069.85	46851.18	2264.26	17086.39	-486.72

227	387.1788 / 9.12	Unknown	387.18	9.13	3909.46	367.49	3271.13	293.44	5043.55	67.68	4702.47	269.54	1134.09	1431.35
228	397.3816 / 16.69	Unknown	397.38	16.69	4077.59	1157.64	2851.42	107.07	4201.56	811.20	3525.87	367.89	123.97	674.46
229	407.3644 / 15.67	Unknown	407.36	15.67	5928.96	598.88	5995.05	447.39	5994.81	317.22	6768.66	81.43	65.85	773.61
230	432.2359 / 9.13	Unknown	432.24	9.13	4535.50	222.42	3914.38	237.58	6132.20	172.49	5577.48	153.86	1596.70	1663.10
231	435.3566 / 15.81	Unknown	435.36	15.83	8818.44	1374.77	9730.21	281.16	8622.08	1201.97	8425.87	670.71	-196.36	-1304.34
232	442.2834 / 7.79	Unknown	442.28	7.80	0.00	0.00	0.00	0.00	23321.30	4132.66	20705.02	2378.45	23321.30	20705.02
233	442.7854 / 7.77	Unknown	442.79	7.78	0.00	0.00	0.00	0.00	12357.94	2221.55	12046.54	1129.50	12357.94	12046.54
234	454.2896 / 11.16	Unknown	454.29	11.15	32550.08	1541.06	39907.09	2004.43	33224.54	866.27	42408.92	1100.66	674.46	2501.83
235	457.3385 / 15.81	Unknown	457.34	15.83	6106.39	724.72	6437.08	287.26	6351.56	723.59	6256.15	521.97	245.17	-180.93
236	468.3058 / 11.03	Unknown	468.31	11.03	45823.98	1851.21	96046.48	4280.99	45511.73	1855.84	97816.97	1494.63	-312.24	1770.49
237	468.4389 / 13.71	Unknown	468.44	13.72	12184.73	2401.36	14105.67	543.16	12522.94	1899.36	11737.29	788.35	338.21	-2368.38
238	469.3831 / 14.75	Unknown	469.38	14.76	7382.50	1437.97	8683.78	268.51	7249.58	1097.43	7277.33	395.69	-132.92	-1406.46
239	474.2805 / 7.54	Unknown	474.28	7.54	0.00	0.00	0.00	0.00	3206.87	609.13	3509.74	581.40	3206.87	3509.74
240	478.2895 / 10.84	Unknown	478.29	10.84	52519.72	1996.01	81609.69	2705.65	51012.27	593.69	86500.22	1623.77	-1507.44	4890.53
241	496.3368 / 12.31	Unknown	496.34	12.33	432235.27	14024.36	540243.67	17082.41	429869.70	9813.66	571752.30	19178.13	-2365.56	31508.63
242	497.2169 / 5.35	Unknown	497.22	5.35	3674.17	250.20	5664.18	170.08	0.00	0.00	94.32	9.46	-3674.17	-5569.86
243	497.3424 / 12.34	Unknown	497.34	12.32	95186.30	5288.84	113554.49	3726.02	93390.47	1704.84	120672.43	2941.52	-1795.83	7117.93
244	513.4089 / 14.71	Unknown	513.41	14.71	7554.19	1350.98	9191.70	410.44	8281.28	1263.99	8162.66	418.23	727.09	-1029.05
245	515.3164 / 11.40	Unknown	515.32	11.40	10209.69	438.42	16893.37	206.26	11546.05	257.46	19615.73	734.66	1336.36	2722.36
246	520.3363 / 11.83	Unknown	520.34	11.83	328350.79	10376.69	440935.58	20489.67	319864.70	9448.40	457768.81	23974.30	-8486.09	16833.23
247	521.3410 / 11.83	Unknown	521.34	11.82	77386.12	2447.72	107167.68	4190.10	73186.04	1855.06	102200.10	4704.83	-4200.07	-4967.58
248	522.3521 / 12.62	Unknown	522.35	12.62	80848.36	4068.41	85108.98	733.69	82333.37	2008.44	96826.79	2963.97	1485.01	11717.81
249	524.3695 / 13.78	Unknown	524.37	13.78	31950.98	2040.22	29401.62	2592.64	30815.79	623.34	27041.41	1421.31	-1135.19	-2360.22
250	536.1618 / 17.57	Unknown	536.16	17.57	5007.08	1617.22	2605.38	268.46	9252.41	6141.38	1692.26	142.21	4245.34	-913.12
251	543.1322 / 0.97	Unknown	543.13	0.98	37410.45	800.98	14238.47	546.86	30099.40	932.14	17356.96	961.40	-7311.04	3118.49
252	569.4326 / 17.37	Unknown	569.43	17.37	8266.57	495.66	9116.62	573.97	8648.18	546.49	7368.83	1608.65	381.61	-1747.79
253	575.5011 / 15.29	Unknown	575.50	15.28	28461.69	3667.11	30852.66	3460.14	15075.46	1916.53	16618.72	1686.33	-13386.23	-14233.94
254	575.5011 / 17.40	Unknown	575.50	17.42	16742.60	4402.46	14165.49	1881.36	14833.23	3258.24	13035.26	1317.83	-1909.37	-1130.24
255	577.5162 / 15.99	Unknown	577.52	16.00	8316.10	2404.94	6296.68	708.70	5417.84	1208.48	5078.73	491.78	-2898.26	-1217.94
256	588.4439 / 12.98	Unknown	588.44	12.98	40189.86	6816.95	43760.65	1772.29	37198.24	4860.33	33679.07	2526.14	-2991.63	-10081.59
257	596.5061 / 14.61	Unknown	596.51	14.62	10274.70	1812.50	11475.66	515.17	9559.43	1315.16	8846.38	383.19	-715.26	-2629.27
258	601.5159 / 17.47	Unknown	601.52	17.47	15333.63	3346.40	12382.68	1312.35	14730.07	2456.51	15109.52	1583.55	-603.56	2726.84
259	610.1826 / 18.21	Unknown	610.18	18.22	6250.70	2929.03	2746.21	113.16	4254.20	1500.55	1822.69	174.80	-1996.50	-923.51
260	613.4777 / 15.01	Unknown	613.48	15.04	7065.28	260.77	11604.15	944.18	1813.72	215.53	2476.08	378.73	-5251.57	-9128.07
261	613.4793 / 14.08	Unknown	613.48	14.08	1965.70	69.88	4270.74	418.31	516.59	34.29	750.92	73.31	-1449.11	-3519.82
262	615.4929 / 17.42	Unknown	615.49	17.41	15640.65	1881.63	14579.59	862.73	15464.37	931.90	15905.29	703.35	-176.29	1325.70
263	626.3089 / 6.08	Unknown	626.31	6.08	4249.67	225.07	4538.16	274.93	127.56	13.66	145.36	12.03	-4122.11	-4392.80
264	632.4690 / 12.92	Unknown	632.47	12.93	31804.75	5383.54	35162.24	1483.30	30163.25	4058.53	27590.79	1937.79	-1641.50	-7571.45
265	639.4913 / 17.11	Unknown	639.49	17.12	18114.94	2082.47	16606.18	631.25	19022.29	2044.56	20162.61	1267.92	907.34	3556.43
266	641.5087 / 17.49	Unknown	641.51	17.47	18966.12	1532.64	18915.72	1319.08	18903.77	1023.05	21016.40	1053.31	-62.35	2100.68
267	651.3603 / 10.62	Unknown	651.36	10.63	21724.95	1404.02	23543.47	2095.78	331.89	95.06	161.00	28.96	-21393.06	-23382.47
268	663.4522 / 17.81	Unknown	663.45	17.81	210647.37	27641.27	232054.26	5006.84	204868.99	23583.92	193354.13	11055.68	-5778.38	-38700.13
269	664.4532 / 17.82	Unknown	664.45	17.79	84919.90	7499.80	96908.14	4057.87	85410.72	10337.49	79065.77	2588.52	490.82	-17842.38
270	676.4948 / 12.90	Unknown	676.49	12.91	20611.50	3490.42	23014.43	955.40	20227.86	2665.72	18274.52	1289.80	-383.64	-4739.91
271	677.3679 / 10.65	Unknown	677.37	10.65	1767.18	16.17	4772.88	425.64	3496.90	131.29	10571.41	688.12	1729.72	5798.53
272	681.4929 / 14.15	Unknown	681.49	14.15	9568.19	979.34	10728.30	461.46	8704.61	896.74	7738.32	409.37	-863.58	-2989.98
273	685.4296 / 17.76	Unknown	685.43	17.80	52724.65	5129.05	61138.35	964.47	56022.96	5370.29	58743.13	2178.59	3298.31	-2395.23
274	701.3672 / 10.40	Unknown	701.37	10.41	3367.41	95.20	12156.66	381.08	4355.85	89.74	14798.82	423.96	988.44	2642.16
275	703.5061 / 14.12	Unknown	703.51	14.13	7812.50	848.12	8774.37	392.34	6757.43	720.04	5996.59	271.73	-1055.07	-2777.78
276	716.5196 / 17.12	Unknown	716.52	17.13	51971.63	13260.25	62135.61	9378.22	16539.28	4004.34	18559.85	3152.61	-35432.35	-43575.76
277	717.4523 / 14.94	Unknown	717.45	14.94	4064.68	1177.93	6356.21	358.05	4489.02	529.44	4411.79	647.19	424.34	-1944.42
278	722.4083 / 14.93	Unknown	722.41	14.94	3620.97	908.44	4997.18	176.68	4680.83	459.00	5044.25	555.60	1059.86	47.06
279	738.5010 / 15.74	Unknown	738.50	15.76	3878.26	822.84	8104.20	1599.29	694.04	43.89	1294.14	183.99	-3184.22	-6810.07
280	740.5191 / 16.33	Unknown	740.52	16.36	36317.15	7368.22	39790.28	5598.98	15222.13	1946.51	21697.24	2267.52	-21095.02	-18093.04

281	758.5639 / 16.37	Unknown	758.56	16.40	141209.54	3931.27	184308.13	15382.49	34048.91	11366.56	59595.21	12088.40	-107160.63	-124712.92
282	760.5800 / 15.96	Unknown	760.58	15.77	43127.17	4319.50	51953.01	9138.03	44776.18	820.36	33117.99	4713.53	1649.01	-18835.02
283	780.5503 / 16.51	Unknown	780.55	16.51	28924.60	745.51	39011.92	3477.10	10213.37	1791.01	13630.26	2285.04	-18711.23	-25381.66
284	784.5785 / 17.11	Unknown	784.58	17.12	27415.20	191.98	36533.50	2062.95	17477.59	4171.22	27891.69	966.30	-9937.61	-8641.81
285	792.5927 / 15.57	Unknown	792.59	15.58	5454.03	794.14	3878.10	484.90	5772.75	308.73	4047.92	264.54	318.72	169.82
286	797.5137 / 15.01	Unknown	797.51	15.03	10514.46	430.75	15586.27	978.80	2885.26	352.07	4001.87	601.81	-7629.20	-11584.40
287	86.0967 / 1.28	Unknown	86.10	1.30	2985.36	336.36	7206.34	831.95	2715.15	429.33	3380.01	322.60	-270.22	-3826.33
288	939.5964 / 15.11	Unknown	939.60	15.12	3899.50	282.87	6507.34	217.04	3946.60	299.30	6713.22	484.40	47.10	205.88
